# Acquired von Willebrand syndrome in children

**DOI:** 10.1016/j.rpth.2025.103169

**Published:** 2025-09-03

**Authors:** Emmanuel J. Favaloro, Leonardo Pasalic

**Affiliations:** 1 Haematology, Sydney Centres for Thrombosis and Haemostasis, Institute of Clinical Pathology and Medical Research, New South Wales Health Pathology, Westmead Hospital, Westmead, New South Wales, Australia; 2 Sydney Centres for Thrombosis and Haemostasis, Research and Education Network, Westmead Hospital, Westmead, New South Wales, Australia; 3 Faculty of Science and Health, Charles Sturt University, Wagga Wagga, New South Wales, Australia; 4 School of Medical Sciences, Faculty of Medicine and Health, University of Sydney, Westmead Hospital, Westmead, New South Wales, Australia; 5 Westmead Clinical School, University of Sydney, Westmead Hospital, Westmead, New South Wales, Australia

von Willebrand disease (VWD) is reportedly the most common bleeding disorder but is potentially both underdiagnosed and misdiagnosed [1,2]. VWD is caused by a deficiency and/or defect in the adhesive plasma protein, von Willebrand factor (VWF). There are 6 types of VWD, defined according to the level and activity (or associated defect) in VWF [3]. Congenital VWD is primarily caused by genetic changes in the *VWF* gene. Acquired forms of VWD also exist and are usually called acquired von Willebrand syndrome (AVWS). AVWS may arise in a variety of disorders [2]. For example, a type 1–like AVWS can occur in hypothyroid conditions due to decreased protein production.

Other forms of AVWS can occur due to absorption of plasma VWF onto activated or abnormal platelets or onto malignant cells, and this can occur in several cancers [2]. Additional forms of AVWS can occur due to antibody-mediated clearance of plasma VWF (eg, in myeloma) or absorption of VWF onto foreign surfaces (eg, mechanical heart valves), or mechanical destruction of VWF under high shear stress (eg, aortic stenosis) [2,4,5].

The diagnosis of VWD or even AVWS may be particularly challenging in children. For VWD, children have generally not had many hemostatic challenges, and thus a personal history of bleeding is incomplete. For AVWS, many of the primary AVWS-associated disorders are age related and have yet to occur in children. Even conditions that can occur in both adults and children may present differently in adults and children.

In a recent issue of this journal, Trębacz et al. [5] focused on AVWS in young children with congenital heart defects, with a particular focus on patent ductus arteriosus (PDA) and ventricular septal defects (VSD). These conditions cause blood shunting between systemic and pulmonary circulation, increasing shear stress, and, in this way, may contribute to AVWS. Their study involved 54 children with PDA and VSD, and the authors performed VWF ristocetin cofactor (VWF:RCo) activity, VWF collagen binding (VWF:CB), and VWF antigen (VWF:Ag) to identify patients with a reduced VWF:RCo/Ag and/or a VWF:CB/Ag ratio, as a possible marker of AVWS, with these patients also undergoing VWF multimer analysis. The authors then calculated a VWF large multimer index (LMI) as an additional test parameter.

Of the 54 patients, 26 (48.1%) fulfilled the criteria for multimer analysis, and an AVWS-like phenotype was found in 13 (24.1%). These patients had significantly lower high-molecular-weight multimers and VWF-LMI than the other patients. A VWF-LMI <0.8 effectively predicted an AVWS-like phenotype with a sensitivity of 1.0 and a specificity of 0.87. Interestingly, this utility was followed by that of the VWF:CB/VWF:Ag ratio, with a sensitivity of 0.57 and specificity of 0.80 at the same cutoff. In contrast, the VWF:RCo/Ag was less useful as a marker of AVWS-like phenotype. The authors concluded that nearly a quarter of children with VSD and PDA may exhibit an AVWS-like phenotype and that the VWF:CB/VWF:Ag ratio is potentially suitable for screening in this group, as well as VWF multimer analysis and VWF-LMI assessment (which is typically less readily or rapidly available).

This study raises several considerations. First, not all VWF activity assays are equal in terms of utility for diagnosis of VWD or AVWS, nor are they available in all geographic localities [6]. First, the VWF:RCo assay has been criticized for its high assay variability, leading to several guidelines recommending use of more modern alternatives to VWF:RCo, such as those based on recombinant glycoprotein (GP) Ib (ie, VWF:GPIbR) or recombinant gain-of-function mutations for GPIb (ie, VWF:GPIbM) [7,8]. However, even these more modern alternatives for VWF:GPIb binding (VWF:GPIbB) assays show considerable variability on a case-by-case basis, as well as low-level detection sensitivity issues, and variation in sensitivity to different type 2 VWD mutations [9,10]. The VWF:CB assay also shows variability in utility between different enzyme-linked immunosorbent assay (ELISA) based assays and the chemiluminescence immunoassay (CLIA)-based procedure. In our own prior evaluations of laboratory test practice, the CLIA-based VWF:CB activity assay clearly surpassed all alternative VWF:GPIbR, VWF:GPIbM, and VWF:RCo activity assays in terms of lowest variability, best low-level VWF sensitivity, and least diagnostic errors in an external quality assessment setting [9]. The CLIA-based VWF:CB and VWF:GPIbR assays have also been shown to express a high correlation to VWF multimer patterns in a separate analysis [11]. Therefore, to some extent, some findings from Trębacz et al. [5], notably better performance of the CLIA-based VWF:CB compared with the platelet-based VWF:RCo, could have been predicted based on such historical data. Nevertheless, the study provides a nice confirmation on the specific utility of the CLIA-based VWF:CB.

Different VWF activity assays have preferential application in different geographical locations [6]. In Australia, where the authors are based, VWF:CB testing is used by approximately 50% of laboratories undertaking testing for diagnosis or exclusion of VWD [9]. Moreover, the CLIA-based VWF:CB is being increasingly adopted as a replacement for ELISA-based VWF:CB assays, as are VWF:GPIbR and VWF:GPIbM assays as replacements for classical VWF:RCo assays [9]. In contrast, VWF:RCo remains dominant in developing countries, in part because of cost and availability issues for the more modern alternatives [6]. In North America, VWF:CB testing is hardly used (<10% of laboratories), and the predominant activity assays are VWF:RCo, VWF:GPIbM, and another assay based on a monoclonal antibody (VWF:Ab), since these are the only assays that are cleared for use by the US Food and Drug Administration (FDA) [12]. In Europe, the situation largely follows that in Australia, due to a similar regulatory framework [13,14].

The findings from Trębacz et al. [5] also have relevance to other conditions leading to AVWS in children, for example, in cardiac assist or mechanical circulatory support, especially for extracorporeal membrane oxygenation (ECMO) [15,16]. ECMO is being increasingly used, including for children, and thus, more patients may be diagnosed with AVWS associated with ECMO use, since VWF, especially high-molecular-weight multimers of VWF, are removed from circulation upon initiation of ECMO [[16], [17], [18], [19]]. Thus, VWF assays, and ratios of VWF activity/Ag, may have clinical utility not only for identification of AVWS in ECMO but also for monitoring of their clinical status during continued ECMO.

In conclusion, not all VWF activity assays are the same, and the use of certain assays is more favorable than those of other assays [[6], [7], [8]]. However, access to different VWF activity assays is geographically based, and both developed countries (based on the regulatory landscape) and developing countries (due to cost and marketed test availability) may not always have access to the best assays for use in diagnosis or exclusion of either congenital VWD or AVWS [6]. The Table provides a summary of the different assays potentially used to diagnose or exclude VWD and AVWS. Despite current guidelines [7,8] recommending a preference for VWF:GPIbR and VWF:GPIbM over VWF:RCo, a well-performed VWF:RCo still has a place in VWD and AVWS diagnostics [6]. Despite current guidelines [7,8] continuing to relegate VWF:CB assays to a second-line position in their diagnostic algorithms, a good VWF:CB assay is invaluable in VWD and AVWS diagnostics [20], and indeed, evidence from Australasia indicates that the CLIA-based VWF:CB and VWF:GPIbR assays outperform the VWF:RCo and VWF:GPIbR and VWF:GPIbM by agglutination, in the arena of VWD diagnostics [9]. Accordingly, the most useful recommendation is that laboratories use the best assays available to them in their geographic locality [6].

**Table:** Summary of the main tests used in the investigation of von Willebrand disease and acquired von Willebrand syndrome.

Test	Abbreviation	What the test measures	Commentary
Factor (F)VIII coagulant activity	FVIII:C	The level of functional FVIII; usually by 1-stage clotting assay based on a modified aPTT; sometimes by chromogenic assay (several manufacturers/suppliers).	Recommended as frontline assay by the authors and current guidelines [[6], [7], [8]]
VWF antigen	VWF:Ag	The level of VWF (both functional and not); historically by ELISA, now mostly by LIA (several manufacturers/suppliers); sometimes by CLIA (1 manufacturer/supplier).	Recommended as frontline assay by the authors and current guidelines [[6], [7], [8]]
VWF glycoprotein Ib binding activity	VWF:GPIbB	Various methods (see further)	Recommended as frontline assay by the authors and current guidelines [[6], [7], [8]]
VWF ristocetin cofactor	VWF:RCo	A VWF:GPIbB assay performed using platelets and ristocetin to measure platelet agglutination (several manufacturers/suppliers); historically, the original VWF activity assay.	Most widely used VWF:GPIbB assay in developing countries and in North America. Acceptable alternative to more modern methodologies for VWF:GPIbB, especially if other assays not available or cost prohibitive.
VWF GPIb binding using recombinant GPIb	VWF:GPIbR	A VWF:GPIbB assay performed using latex or magnetic particles, recombinant GPIb, and ristocetin to measure latex agglutination or chemiluminescence (CLIA)-based events (1 manufacturer/supplier), respectively; a modern alternative to VWF:RCo.	Modern alternative to VWF:RCo preferred as frontline VWF:GPIbB assay by current guidelines [7,8].
VWF GPIb binding using recombinant mutated GPIb	VWF:GPIbM	A VWF:GPIbB assay performed using latex (commercial method; 1 manufacturer/supplier) or ELISA (not yet commercialized), recombinant mutated gain-of-function GPIb (but no ristocetin) to measure latex agglutination or ELISA color generation, respectively; another modern alternative to VWF:RCo.	Modern alternative to VWF:RCo preferred as frontline VWF:GPIbB assay by current guidelines [7,8].
VWF collagen–binding activity	VWF:CB	Primarily performed by ELISA (a large number of manufacturers/suppliers) and increasingly by CLIA (1 manufacturer/supplier). We use the latter and include this test in our first-line panel.	Recommended as frontline assay by the authors [6] but secondline assay by current guidelines [7,8].
VWF FVIII–binding activity	VWF:FVIIIB	Primarily performed by ELISA (1 manufacturer/supplier; or using in-house/laboratory-developed methods).	Recommended by the authors and current guidelines [[6], [7], [8]] for diagnosis or exclusion of type 2N VWD.
Ristocetin-induced platelet aggregation/agglutination	RIPA	Performed by platelet agglutination/aggregation (1 manufacturer of ristocetin, but distributed by several suppliers).	Recommended by the authors and current guidelines [[6], [7], [8]] for identification or exclusion of type 2B or platelet type VWD.
VWF multimers	VWF:mult	Performed by agarose gel electrophoresis (1 commercial semiautomated method; otherwise, in-house/laboratory-developed methods).	Recommended by the authors and current guidelines [[6], [7], [8]] for use in select cases where loss of HMW VWF may occur or to help distinguish select type 2 VWD cases (eg, 2A, 2B, 2M, or platelet type VWD; AVWS).
VWF activity/Ag ratios	VWF:Act/Ag (various ratios as given further below)	These act as surrogate markers for the loss of HMW and intermediate-molecular-weight VWF, but utility depends on specific assays and methodology used. For example, CLIA-based VWF:Act/Ag ratios are the most useful markers of HMW VWF loss. Differential ratios also useful for distinguishing different types of VWD; for example, low VWF:Act/Ag ratios for all VWF activity assays will occur in VWD and AVWS associated with loss of HMW VWF. In contrast, low vs normal VWF:GPIbB/Ag and VWF:CB/Ag ratios suggest 2M VWD.	Recommended in frontline investigations of VWD and AVWS by the authors and current guidelines [[6], [7], [8]], as per recommendations for specific assays used in frontline or second-line test panels. Thus, for guidelines [7,8], this would be VWF:GPIbR/Ag or VWF:GPIbM/Ag as preferred, and VWF:RCo/Ag and VWF:CB/Ag as alternative or second-line testing. For the authors, this would be VWF:GPIbR/Ag and VWF:CB/Ag using CLIA as first-line testing.
	VWF:RCo/Ag		
	VWF:GPIbR/Ag		
	VWF:GPIbM/Ag		
	VWF:CB/Ag		

Ag, antigen; aPTT, activated partial thromboplastin time; AVWS, acquired von Willebrand syndrome; CLIA, chemiluminescence immunoassay; LIA, latex immunoassay; ELISA, enzyme-linked immunosorbent assay; GP, glycoprotein; HMW, high-molecular-weight; RCo, ristocetin cofactor; VWF, von Willebrand factor.
